# The views of key stakeholders in Zimbabwe on the introduction of postgraduate family medicine training: A qualitative study

**DOI:** 10.4102/phcfm.v9i1.1469

**Published:** 2017-09-12

**Authors:** Cherifa Sururu, Robert Mash

**Affiliations:** 1 Division of Family Medicine and Primary Care, Stellenbosch University, South Africa

## Abstract

**Background:**

Strengthening primary health care (PHC) is a priority for all effective health systems, and family physicians are seen as a key member of the PHC team. Zimbabwe has joined a number of African countries that are seriously considering the introduction of postgraduate family medicine training. Implementation of training, however, has not yet happened.

**Aim:**

To explore the views of key stakeholders on the introduction of postgraduate family medicine training.

**Setting:**

Key academic, governmental and professional stakeholders in Zimbabwean health and higher education systems.

**Method:**

Twelve semi-structured interviews were conducted with purposively selected key stakeholders. Data were recorded, transcribed and analysed using the framework method.

**Results:**

Anticipated benefits: More effective functioning of PHC and district health services with reduced referrals, improved access to more comprehensive services and improved clinical outcomes. Opportunities: International trend towards family medicine training, government support, availability of a small group of local trainers, need to revise PHC policy. Anticipated barriers: Family medicine is unattractive as a career choice because it is largely unknown to newly qualified doctors and may not be recognised in private sector. There is concern that advocacy is mainly coming from the private sector. Threats: Economic conditions, poor remuneration, lack of funding for resources and new initiatives, resistance from other specialists in private sector.

**Conclusion:**

Stakeholders anticipated significant benefits from the introduction of family medicine training and identified a number of opportunities that support this, but also recognised the existence of major barriers and threats to successful implementation.

## Introduction

The World Health Organization is committed to the strengthening of primary health care (PHC) ‘now more than ever’ and has recommended four key reforms to achieve this: a more person-centred approach, a more community-orientated approach, more effective leadership and universal coverage.^1^ Effective PHC is also characterised by its ability to provide access, continuity of care, coordination of care and comprehensive services.^2^ The World Health Assembly has recommended that a multidisciplinary team is required to deliver effective PHC that includes primary care nurses, midwives, allied health professionals, family physicians (FPs) and community health workers.^3^ The FP is a doctor with postgraduate training in the field of general practice or family medicine.

Health systems in the African context of low-income and middle-income countries have begun to recognise the need for a FP in the PHC team.^4^ A few countries, such as South Africa and Nigeria, have been training FPs for some time, but many countries are just beginning to do so, such as Botswana and Malawi. One of the key issues has been the realisation that the roles and competencies required of the African FP in the health system are not the same as in high-income countries, which have historically shaped the discipline.^5^ African FPs, for example, will not typically provide first contact care in the public sector, which is usually provided by nurses or mid-level doctors. African FPs must also have additional competencies to work in the district hospital and support PHC teams. Six roles for the African FP in the district health system have been defined: a competent clinician, a consultant to the health care team, a capacity builder and clinical trainer, a leader of clinical governance and champion of a community-orientated approach.^6,7^

There is good evidence from high-income countries for the impact of primary care physicians on health outcomes, and there is also evidence for the effectiveness of PHC teams that include a primary care doctor in middle-income countries such as Brazil.^4^ The WHO also affirms the need for FPs with postgraduate training in effective PHC systems.^1^ In South Africa, studies on the impact of FPs suggest that they have a greater impact across their six roles than doctors without postgraduate training and may currently be making more of a difference in district hospitals than in PHC.^8,9^ District managers report a useful impact on clinical processes as well as health system performance.^10^

Although many countries in Africa are now embracing family medicine training, there are still a wide variety of views amongst stakeholders on the contribution of FPs to health systems and variability in the development of the discipline from countries with no apparent interest to those that have employed FPs widely in the district health services (DHS).^11^ Differences between countries may be because of different political commitments to PHC, different health budgets and economic constraints, misunderstandings about the roles of FPs, poor visibility of the discipline and uncertainty about the evidence for making a clear commitment in policy. Three key stakeholder groups that influence the introduction of postgraduate family medicine training in African countries have been identified as the department of health, the universities or educational institutions, and professional organisations such as the registration council.^12^ Key stakeholders in Zimbabwe have been discussing the implementation of training in family medicine for several years and appear to be moving towards a commitment to action.

In an attempt to advocate and lobby for the introduction of family medicine, the College of Primary Health Care Physicians of Zimbabwe (CPCPZ) held a stakeholder’s workshop in 2012 that conceptualised what this might mean in the Zimbabwean context and aimed to introduce training by 2014. It was noted that family medicine was emerging and developing rapidly in the region, but it was emphasised that the international and regional examples were supposed to be used to develop a contextually suitable programme for Zimbabwe. However, integrating these experiences and the input of the local stakeholders into an appropriate model for Zimbabwe has remained a challenge. Within the CPCPZ, one of the most difficult issues to handle was how to include doctors with previous qualifications or years of experience into the new discipline of family medicine. Apart from the workshop in 2012, there has been no other formal evaluations of the views of key stakeholders on family medicine in Zimbabwe. Understanding such viewpoints may assist with resolving any remaining ambivalence and with the planning of effective implementation.

The aim of the study was to explore the views of key academic, government and health profession leaders in Zimbabwe on the introduction of postgraduate training in family medicine.

## Methods

### Study design

This was a phenomenological qualitative study that used semi-structured interviews to explore the views of key stakeholders in Zimbabwe.

### Setting

The Ministry of Health’s (MoH) mission is to provide, administer, coordinate, promote and advocate for the provision of equitable, appropriate, accessible, affordable and acceptable quality health services and care to Zimbabweans, while maximising the use of available resources, in line with the PHC approach.^13^ Post independence, the Government of Zimbabwe (GoZ) invested heavily in PHC through, for example, the development of community health workers, PHC infrastructure and training of competent health professionals.^14^ As a result, Zimbabwe recorded good progress in family planning, maternal and child health in the period 1980–1995. Thereafter, economic challenges, loss of skills and the HIV pandemic affected the health service delivery system and progress could not be maintained. Women and children, being more vulnerable, have been the most affected by the deterioration of some aspects of the health system.^14^

At the time of the study, CPCPZ was working closely with Stellenbosch University in South Africa and was partnered with the University of Zimbabwe’s College of Health Sciences (UZCHS), with the full support of the GoZ, to introduce family medicine training in Zimbabwe. CPCPZ are a professional body for primary care physicians and the majority of its members are in private practice. This partnership saw a cohort of FPs being trained at Stellenbosch University and by 2017 four had graduated. The Medical and Dental Practitioners Council of Zimbabwe (MDPCZ) opened a new register for family medicine and these four were successfully registered, marking the entry of family medicine in Zimbabwe.

All of the above organisations are considered key stakeholders in the provision of PHC and introduction of family medicine in Zimbabwe. In addition, the central hospitals, Health Services Board (HSB), DHS and Zimbabwe Medical Association (ZiMA) are key stakeholders.

The researcher undertook this study as part of the Master’s of Medicine in Family Medicine at Stellenbosch University. He is amongst the Zimbabwean pioneers to undertake studies in family medicine at Stellenbosch University. At the time of the study, he was part of the lobbying and advocacy group for training in Zimbabwe.

### Sampling and selection

Criterion-based purposeful sampling was used to identify eight interviewees, one from each of the stakeholder organisations (MoH, CPCPZ, UZCHS, MDPCZ, central hospitals, HSB, DHS and ZiMA). This entailed purposively selecting senior individuals in each organisation who were likely to be ‘information-rich’ and able to engage with the aim of the study. Only individuals who had been in their post for the last five years were eligible for selection. Those who were engaged in postgraduate family medicine training or who had such qualifications were excluded.

After the first eight interviews, a preliminary analysis suggested there was need to perform additional interviews to explore emerging themes in more depth or to clarify issues raised. Snowball sampling was then used to identify four additional interviewees that were likely to shed more light on the emerging themes or explain certain concepts in more detail.

### Data collection

The interview guide (see Appendix 1) reflected the aim of the study and ensured that the participants’ understanding of family medicine, ideas about the contribution of family medicine to the health system and views on training were explored. Open-ended exploratory questions were used with reflective listening, summaries and clarifications where needed. The first three tapes were reviewed by the researcher and supervisor to ensure that the guide was appropriately constructed and that the researcher possessed necessary interviewing skills. The opening question was ‘Please tell me what you know about family medicine, its benefits and any possible concerns that you may have’. Interviews were conducted by the principal researcher from June 2015 to April 2016. Nine interviews were performed by the researcher in the interviewees’ offices, three by cell phone, and each interview lasted between 30 and 60 min. Eleven interviews were performed in English and one was in Shona.

### Data analysis

All interviews were transcribed verbatim by a research assistant and these were then cross-checked against the audiotapes by the researcher. The interview material in Shona was translated into English. ATLAS-ti software was used to assist with the analysis of the data using the framework method. The framework method involved the following steps: familiarisation with the raw data, identifying an index of all the codes and categories to be used, applying the index to all the raw data by annotating all the transcripts with the codes, charting all the data from the codes in one category into a single document and interpreting themes from these charts in terms of the range and strength of opinions, as well as any associations or relationships between themes. Analysis was performed by C.S. under supervision of R.M. who particularly checked the coding index and interpretation of data.

### Ethical considerations

The Health Research Ethics Committee of Stellenbosch University (S13/04/057) and the Medical Research Council of Zimbabwe (MRCZ/B/597) provided ethical approval.

## Results

A total of 12 interviews were conducted as shown in Table 1. The researcher was unable to obtain appointments with the Permanent Secretary to the MoH, the Director of HSB and the Dean of the UZCHS.

**TABLE 1:** Profile of responders.

Sector	Number of participants
Zimbabwe Medical Association’s past president	1
College of Primary Health Care Physicians of Zimbabwe national executive members	5
Members of the Medical and Dental Professions Council of Zimbabwe	2
Heads of tertiary hospitals	2
Senior district medical officer	1
Former Health Services board member	1

### Benefits of family medicine to the health system

Almost all respondents believed that a FP would improve the functioning of PHC and the DHS. In particular, they conceptualised that the FP would be better trained than existing health care providers to offer a more comprehensive range of services for a wide variety of health conditions and thereby also reduce referrals to higher levels of care:

‘…[*There*] is a clear benefit in terms of the level of clinical management which is going to be offered in those institutions and hopefully going to cut down on the referrals which are going through to tertiary institutions.’ (Participant 11)

It was conceptualised that greater accessibility to improved care across a wide range of health conditions would result in improved outcomes and reduction of complications. It was, however, also noted that FPs should recognise their limits and refer appropriately:

‘…with good family practice training, morbidity and mortality rates should not be so bad … you should be able to recognise problems before they happen and be able to know how best you can handle them, and … know what you can and cannot do and transfer on time.’ (Participant 7)

### Roles and competencies of the family physician

All respondents expected the FP to be competently trained to deal with most health conditions in the district hospital and the associated PHC clinics, with emphasis on surgical and emergency skills:

‘…the key competencies … in terms of maternal, neonatal and child health; … common conditions that we see. Are they able to look after major road traffic accidents and stabilize those patients before referral to secondary specialists?’ (Participant 10)

The recognition of FPs as specialist by the MDPCZ was seen as putting them on a par with other specialists:

‘There is no register which says you are a specialist but you know you are at sub-specialist or super specialist level. All specialists are at an equal platform as far as council is concerned.’ (Participant 11)

Many of the respondents appreciated that the FP should also serve as a role model and as a resource person to the health care team, including junior medical staff:

‘You don’t learn medicine from a book. You learn theory but you cannot learn how to take the history, how to examine a patient, how to have clinical skills if you don’t see somebody else doing it.’ (Participant 9)

The respondents thought that the FP would take responsibility for supervising and building the capacity of junior medical staff:

‘…making sure the GMOs and other junior medical staff have the right training, identify training they need … adequate CME/CPD activities … the correct … responsibility … basically working efficiently.’ (Participant 12)

Two respondents conceptualised that the FP should lead the district hospital team in saving scarce resources and promoting more efficient care:

‘You instil into these young doctors the issue that resources are finite and they run out, so unless they use them properly so that you stretch their use and impact ….’ (Participant 10)

They were also seen to have a role in corporate governance:

‘…ultimately the doctor is responsible for everything, staff, … costs, … income, expenditure, cost containment ensuring discipline and all those things so all those things he can do or he can delegate some of those things.’ (Participant 12)

Although health education and prevention activities for infectious diseases, which requires community participation, were mentioned by one respondent, none specifically mentioned community-orientated primary care:

‘…I believe that if they get involved with the communities and assist them with other health care personnel and educate the population in preventing diseases, that’s part and parcel of a Family Physician role.’ (Participant 12)

### Concerns with the introduction of family medicine

Two respondents from the regulatory authorities thought that the initiative should have come from the public sector instead of the private sector with the MoH as the main driver instead of CPCPZ:

‘…the way we understand it particularly where they are being trained in South Africa … are primarily being trained and targeted to … public health setting … that is the primary aim … obviously they can also function in the private practice but the initial understanding we have in South Africa is … to strengthen public health care … first and foremost and then … it can then be extended to private practice … but … our pioneer cadres have actually been drawn from private practice. (Participant 11)

Most respondents thought that family medicine was still largely unknown in Zimbabwe and might struggle to attract registrars. This was because it was not taught at undergraduate or postgraduate levels and there were no FPs working in the public health system where they are likely to be more conspicuous:

‘If there is no Family Physician in front of me, there is no way I can imagine being one.’ (Participant 1)

A few respondents thought that in the private sector it may be difficult to obtain recognition for an FP with postgraduate training and a tendency to pay them the same tariff as general practitioners with no postgraduate training, which would limit the attractiveness of the discipline:

‘They might just see you as you haven’t changed, not wanting to upgrade your tariff thinking you are still the same.’ (Participant 1)

### Opportunities for the introduction of family medicine

Despite some concerns, the majority of the respondents were in support of introducing family medicine in Zimbabwe, because it was an international trend with other countries moving towards generalists having postgraduate training:

‘And from a regulatory point of view again we refer to other councils which no longer actually register general practitioners. They have moved away from there because they believe that such cadres must also undergo postgraduate training. No one must be left at a level like a static level of undergraduate … in future we are going to move in that direction where we eliminate the register of general practitioners and everybody is a specialist in whatever field they wish to pursue.’ (Participant 11)

A few respondents felt that the programme had the full support of the GoZ:

‘…we have vowed to give our utmost support that such specialist training takes off sooner rather than later in this country.’ (Participant 11)

Half of the respondents noted that the country had already taken positive strides towards the establishment of family medicine through the recently graduated FPs who could become clinical trainers:

‘…the College needs to look at people who have been through the programme and they are going to say these are the skills that we have learnt and we can teach … either in their practices, … but very importantly, the College needs to get involved in education.’ (Participant 9)

Stakeholders noted the advocacy that had already taken place and the need for ongoing lobbying and support for the implementation of family medicine:

‘I think … people need to understand what Family Medicine is all about … this battle has been going on for some time. …,I don’t expect people to be just in line just like that but we have to convince them, fight for that, even convince the parliamentary committee and it should not be easy but, we shall succeed….’ (Participant 6)

Half the respondents thought that the time had come for Zimbabwe to change how the PHC system was working by incorporating an expertly trained generalist (FP), with the hope of making it more responsive to changes in the health needs of the population:

‘…this is a course designed to meet the new changes in our society, whether you are talking about medicine in general or the way we have to approach surgical cases as a GP.’ (Participant 2)

### Threats to the introduction of family medicine

Some of the respondents conceptualised that the impetus was on the GoZ to overhaul its remuneration system to help attract or retain FPs in remote areas, or allow private practice by FPs it employs:

‘…the impetus then should be for government to provide a salary that doctors can live off to encourage them to stay within the hospital sanctuary system.’ (Participant 9)

‘Government might employ the doctor full time but allow him to do his own private thing at the district centre or at the growth point. That will be another way of doing it.’ (Participant 1)

Most of the respondents wondered how the FPs were going to be retained in the country given the sustained ‘brain drain’ of critical staff because of the deteriorating economy:

‘We have lots of doctors all over in Canada, Australia, Botswana, and Namibia and here we are short of doctors. I recall at one time as a house man in the 80’s in Mpilo we had cardiac surgery being done at Mpilo … they have since gone away….’ (Participant 7)

Almost all respondents thought that enhanced competencies, for example in surgical or obstetric skills, would help the public sector health services. However, in the private sector, the possibility of lower patient flows to the hospital specialists was seen as a possible area of conflict. However, all respondents concurred that mutual respect and working in a complementary way would bring harmony to the medical fraternity:

‘The specialists think you will be intruding into their domain because since you have done some further training in orthopaedics, further training in paediatrics, further training in obstetrics….’ (Participant 1)

‘Our attitude and the way we are going to work will have to change. But they should know we are not competing but complementing them. When we interact we should give each other respect.’ (Participant 1)

A few respondents conceptualised that with the GoZ failing to sustain the existing infrastructure, because of financial limitations, any new policy on family medicine was not going to be resourced:

‘…There are so many things that have been planned and they have not been fulfilled as yet because there are no facilities, resources to follow up.’ (Participant 4)

### Possible training sites

All respondents were in favour of decentralised training with appropriate supervision to ensure the production of quality FPs.

‘…so work based training is now very much in flourish and we already have quite a significant number of specialists who are not actually being trained at the College of Health Sciences they are trained at the various institutions and became specialists and they are being registered by Council.’ (Participant 11)

One respondent from a regulatory authority noted that any academic institution was capable of hosting the FM programme, including the possibility of having the programme run from universities outside the country, as long as the training could be accredited:

‘So there is no longer an issue or a barrier to training that you have got to have your umbilical cord at … or any other local university for that matter.’ (Participant 11)

## Discussion

### Key findings

The key findings were classified into four main categories as shown in Box 1 below: anticipated benefits, anticipated barriers, opportunities and threats to implementation.

BOX 1 Summary of key findings.

**Table:** 

Anticipated benefits	Anticipated barriers
Effective functioning of PHC and DHS with reduced referrals Improved access to more comprehensive services at DHS level Improved clinical outcomes Expert generalists with five identified roles: Specialist care provider Capacity builder and trainer Monitor of resources for efficient use Corporate governance Health education and disease prevention	Initiative coming from the private rather than public sector Family medicine is unknown and may struggle to attract registrars Lack of recognition in the private sector may also be a disincentive
**Possible opportunities** International trend towards family medicine training Government support Availability of a small cadre of local clinical trainers Need to revise the PHC and DHS services	**Possible threats** Poor remuneration in the public sector Harsh economic environment Turf wars with other specialists Lack of resources for implementing new policy

DHS, district health services; PHC, primary health care.

### Discussion of key findings

The stages of change model, as applied to the implementation of family medicine training in Africa, suggests that Zimbabwe is at the contemplation stage, but moving towards action.^12^ The results suggest that the main stakeholders are all now behind the implementation of family medicine, although not all of them could be interviewed directly.

Key factors elsewhere that support implementation have been identified as the opening of a new medical school, the role of individual champions, the role of informal training programmes in creating an initial cadre of FPs and organisations advocating for family medicine.^12^ This study identified many of these supportive factors with strong individual champions and organisational advocacy coming from the CPCPZ. Although the study identified the potential of a small cadre of locally trained FPs, more experienced academic FPs are lacking and it is likely that programmes will initially be situated in community health departments. Community health specialists may not fully understand the requirements for training of FPs. Many countries have made use of senior FPs trained in other countries to initiate their programmes, although care must be taken to ensure that appropriate models of family medicine are promoted.^12^ FPs from high-income countries may also lack the necessary district hospital skills.

Key factors elsewhere that hinder implementation have been identified as a lack of policy commitment to PHC, confusion about the nature of family medicine and role of the FP as well as resistance from other specialists.^12^ This study suggests that Zimbabwe intends to revitalise its policy commitment to PHC, retains some confusion about the nature of family medicine and a superficial understanding of the role of the FP. Some resistance from specialists, particularly in the private sector, may also be expected. Although the respondents identified most of the roles that have been defined in other African countries, they did not have a clear understanding of the role of consultant, leader of clinical governance or supporter of community-orientated primary care. On the contrary, they emphasised a managerial role in corporate governance, which has been rejected in some other countries.^15^ It is thought that FPs should primarily be clinicians and not managers. It is possible that respondents extrapolated their ideas of what the FP would do by combining their experience of the current roles of the general medical officer in district hospitals with the more managerial roles of the medical superintendent.

Factors identified that were more unique to the Zimbabwean context included the severe economic environment and role of the private sector in promoting family medicine. The lack of financial resources threatens the launch of new training programmes and the creation of posts in the public sector for registrars, faculty and FPs. Currently many doctors, even if working in the public sector, are reliant on earnings from the private sector to survive, because salaries are very low. This would also be a disincentive for disseminated training sites as opportunities for private practice are greater in urban areas. CPCPZ is also regarded with some suspicion by the public sector as its members are largely from the private sector. In the longer term, however, strengthening PHC is likely to develop a more cost-effective health system.

Although the respondents appeared in favour of disseminated training within the DHS, they did not appear to have considered all of the obstacles. A similar study in rural Botswana identified key issues such as personal lifestyle challenges (difficulty with housing, travel, electricity, clean water, suitable schools and good quality food), a lack of other human resources and infrastructure to create a suitable learning environment and a lack of suitably qualified supervisors.^16^ Training FPs in the DHS is an important principle, but sometimes the training programme has to evolve to achieve this.^17^ In South Africa, initial training made more use of regional and referral hospitals, as well as other specialists, before moving more into the districts. Care must also be taken to ensure that the first FPs trained in Zimbabwe are of a high calibre as the discipline will be judged according to their performance.

### Limitations

The researcher (C.S.) was a family medicine registrar at the time of this study, and an advocate for the discipline. Although he did his level best to remain neutral whilst conducting the interviews, this interest could have affected his perception of the views expressed by the respondents. C.S. has worked as a general practitioner with no postgraduate training in family medicine for more than 15 years and has been a member of CPCPZ for more than 10 years. Most of the respondents are known to him and some have worked with him. This fact may have affected the way they expressed their views, despite assurances of confidentiality. Some of the eligible participants could not schedule interviews to participate in the study for undisclosed reasons. They could have provided alternative viewpoints, but their reasons for not scheduling interviews were not known.

### Recommendations

Stakeholders should reach a clearer and more in-depth consensus on the roles and competencies required of FPs in the Zimbabwean health system in order to guide local training programmes.

Stakeholders should create and implement policy for PHC and DHS in Zimbabwe that includes the FP as part of the multidisciplinary team, improves working conditions and provides incentives to attract and retain health workers.

Stakeholders may need to consider a model of training that evolves to the ideal over time. Initial training may need to make use of existing specialists and referral hospitals, while moving towards a more fully disseminated and district-based model of training.

The CPCPZ remains a key stakeholder and should continue to advocate and lobby for family medicine in collaboration with partners from the public sector. The suspicion that the initiative is more orientated towards private practitioners should be addressed.

## Conclusions

Stakeholders believed that family medicine would improve access to more comprehensive PHC services with improved quality of care and clinical outcomes as well as fewer referrals to higher levels of care. Respondents were concerned about poor recognition and remuneration of FPs, the need to work in both private and public sectors to secure a sufficient income, resistance from other specialists competing for business in the private sector, and a lack of a clear vision for PHC in Zimbabwe within which FPs could be located. PHC is also suffering from a lack of infrastructure and resources. The respondents identified key roles for the FP which were similar to other countries in the region, although emphasised a managerial role more than elsewhere. Stakeholders recognised that a revitalisation of policy on PHC and DHS provided an opportunity to conceptualise the role of the FP and that many countries in the region were now embracing postgraduate training in FM. The harsh economic climate in Zimbabwe makes it difficult to create new posts or introduce a new discipline. Training will need to be located within a higher education institution and make use of the few local FPs as trainers. An initial model of small-scale high-quality training with evidence of early impact in one part of the health system may be more persuasive in securing ongoing support.

## Appendix 1

### A qualitative study of the views of key leaders in Zimbabwe on family medicine

#### Opening question

Please tell me what you know about family medicine, its benefits and any possible concerns that you may have.

**The interview guide (with possible prompts below):**

- Can you tell us about family medicine? Benefits? Concerns?
- What are your thoughts on the role of family medicine in Zimbabwe? Currently? Potentially? In the district health services or primary health care?
- What do you think are the issues in implementing the discipline of family medicine? Opportunities? Threats?
- What are critical human resource issues to establishing family medicine?
- What are your views on appropriate training in family medicine?

**Tell me about family medicine and the relationship with other clinical disciplines**

- What other aspects of the health care system are critical to the establishment of family medicine?
- Do you have anything else to add?
